# Annual of the Universal Medical Sciences

**Published:** 1894-01

**Authors:** 


ANNUAL OF THE UNIVERSAL MEDICAL SCIENCES. (Issue of 1893.) Edited by CHARLES E. SAJOUS, M. D., and SEVENTY ASSOCIATE EDITORS. *Assistsd by over Two Hundred Corresponding Editors, Contributors and Correspondents.* Illustrated with Chromo Lithographs, Engravings and Maps. 1893. F. A. Davis Company, Publishers, Philadelphia, New York, Chicago and London. *In Five Octavo Volumes.* About 500 pages each. *Sold only by Subscription.* $15.

This 1893 issue of "yearly report of the progress of the general sanitary sciences throughout the world" is in keeping with its predecessors in thoroughness. Each department of medicine, surgery, obstetrics, anatomy, physiology, etc., has been handled with a masterly hand, and the result is the bringing together of, apparently, all advances that have been made in the respective departments. Indeed it would seem that this work is indispensable to the practitioner—whether special or general—who feels the earnest responsibility of his calling in dealing with human health and life; for it supplements the best of text-books of the most recent date, and tells him of things of value that have been discovered or devised during the year.

It is a moderate annual amount to expend $15 for such a storehouse of information—systematically arranged with excellent indexes, which enable the owner to readily refer to any subject about which advance has been made—especially when that expense also entitles the subscriber to the *Universal Medical Monthly*. A work like this cannot be reviewed. We can only commend it to every medical man as containing the latest and the best of valuable information. E. X.

